# Old cytokine, new tricks: A refined model of interferon’s antiviral activity

**DOI:** 10.1371/journal.pbio.3003154

**Published:** 2025-05-07

**Authors:** John W. Schoggins

**Affiliations:** Department of Microbiology, UT Southwestern Medical Center, Dallas, Texas, United States of America

## Abstract

Interferon is a central component of the vertebrate antiviral immune response, thought to act through induction of hundreds of interferon-stimulated genes, with some redundancy. This Perspective highlights recent findings that suggest a more refined ‘limited set’ model, in which distinct viruses are targeted by small subsets of the induced gene repertoire.

## Teaser

Interferon is a central component of the vertebrate antiviral immune response, thought to act through induction of hundreds of interferon-stimulated genes, with some redundancy. Recent findings suggest a more refined ‘limited set’ model, in which distinct viruses are targeted by small subsets of the induced gene repertoire.

Viruses remain a major public health concern, causing significant disease, economic losses, and strain on healthcare systems worldwide. However, from an evolutionary standpoint, humans and other vertebrates mount surprisingly effective defenses against most viral infections. Central to these defenses is interferon, an antiviral cytokine that evolved over 450 million years ago and is essential for vertebrate survival from viral infections [1,2]. When interferon is produced, it activates the JAK/STAT signaling pathway and induces hundreds of genes—referred to as interferon-stimulated genes (ISGs)—many of which encode proteins with unique antiviral mechanisms [3].

**Fig 1:**
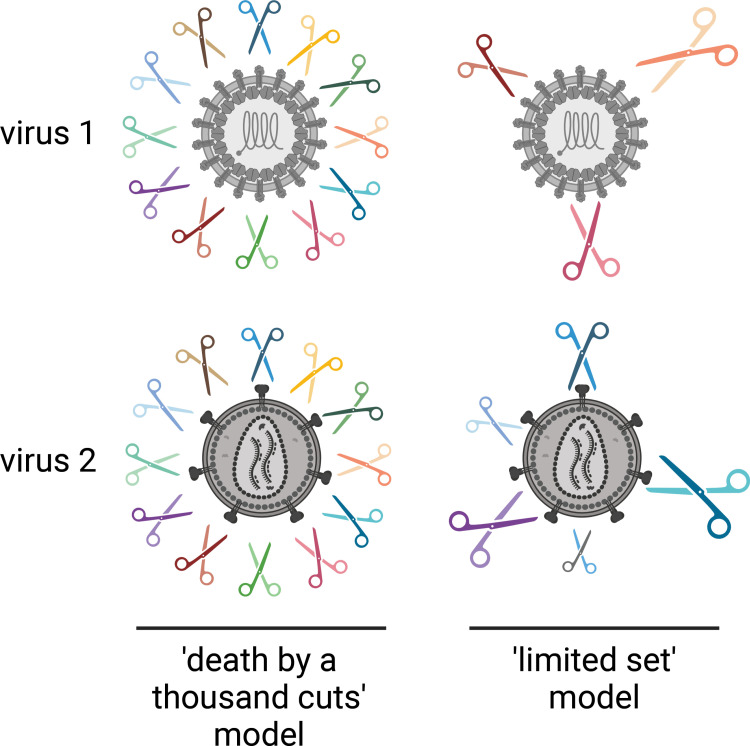
A refined model of interferon action. The conventional ‘death by a thousand cuts’ model describes how many minor interferon-stimulated genes (ISGs) act together to suppress viral replication. The new ‘limited set’ model contrasts this and posits that a limited set of dominant ISGs make up the majority of the IFN-mediated antiviral effect. The system is likely tailored so that distinct sets of genes may target different viruses. Figure generated with Biorender.

Although interferon was discovered in 1957 [4], it took decades of research, including large-scale functional genetic screens starting in 2011, to begin identifying precisely which ISGs directly inhibit viruses [5]. However, a critical question posed for nearly 40 years has persisted: how many ISGs are required to protect against a given virus [6]? A prevailing view is that many, perhaps dozens, of ISGs combine to produce interferon’s full antiviral effect; the so-called “death by a thousand cuts” model (Fig 1). This model likely emanated from genome-scale transcriptomics studies showing that interferon induces many hundreds of ISGs [7]. Logically, such a brute-force transcriptional response would include dozens of antiviral effectors working together to curtail viral replication. However, recent findings suggest a different scenario, one that relies on widespread induction of ISGs, but only a few of these genes drive most of the antiviral effect [8,9]. This “limited set” strategy ensures that for any given virus, the system has a broad array of options, with only a few ISGs proving indispensable in each case.

To determine how many individual ISGs are required for interferon-mediated protection, our group performed two complementary knockout-based CRISPR screens: a genome-wide screen and an ISG-focused screen in human cells [8]. In each approach, we deleted a single gene per cell, treated the cells with interferon to establish an antiviral state, and infected them with the alphavirus Venezuelan equine encephalitis virus (VEEV). Infection in an interferon-treated knockout cell indicated that the missing gene was critical for protection.

One would, of course, expect the interferon receptors and signaling molecules in the JAK/STAT pathway to be hits in the screen, as they are required to activate the response. If any ISGs also inhibited the virus, they would appear as hits. The notable finding from our studies was that only three ISGs were shown to inhibit VEEV: IFIT1, IFIT3, and ZAP. All three have been well characterized for their anti-alphavirus activity [10], so they were strong confirmations that our screen worked. What was surprising is that no additional ISGs were significant hits in the screens; we therefore wondered, “are these three genes doing all the work?”.

To test their collective effects, we used combinatorial CRISPR gene knockout, where we silenced all three genes simultaneously. The three-gene knockout significantly impaired interferon’s ability to inhibit VEEV, indicating that the majority of the interferon response was mediated by these genes. This was unexpected because, in the same cells, interferon induces more than 600 genes. The implication is that most of those genes are superfluous and not required for cell-intrinsic restriction of this particular virus. Remarkably, another alphavirus, O’nyong nyong virus, was still robustly suppressed by interferon even when the three critical anti-VEEV ISGs were missing. We still do not know which ISGs inhibit that virus. But, the data suggest that even within the same viral family, the ISG repertoires targeting related viruses may differ.

A similar discovery was made in another study published around the same time [9]. Here, the authors performed a CRISPR-based knockout screen to identify ISGs that inhibit HIV-1 in primary human T cells. They demonstrated, again through combinatorial knockout studies, that five genes dominated the interferon-mediated suppression of HIV-1. Their study also raised the interesting prospect that the relevant gene sets may shift from one strain of HIV-1 to the next, similar to our findings with distinct alphaviruses.

These results suggest that interferon does not rely on massive redundancy for antiviral defense, but rather on a broad induction of ISGs that provides a flexible repertoire (Fig 1). In this “limited set” model, the host cell throws all possible effectors into play, and whichever subset of genes proves effective for a given virus emerges as the dominant defense. But how does this system become so tailored? One possibility is that evolutionary pressures help shape distinct interferon effectors for different viral threats. Many ISGs are rapidly evolving, suggesting they may be under immune pressure to adapt in ways that enhance control of infection [11,12]. Such pressures may favor specialized ISGs, leading to a combinatorial system in which a small set of genes controls any given virus.

Reflecting on earlier research, it is evident that this emerging model has a solid premise. Knockout mouse experiments have shown that deleting a single ISG can profoundly affect an animal’s susceptibility to viral infections, indicating that individual genes can have outsized effects on interferon function [13]. This would not be predicted in a ‘death by a thousand cuts’ model, which would argue for single genes to have relatively minor effects. Removing just a few more genes, as demonstrated by the combinatorial knockout studies described above, nearly neutralizes interferon. Thus, while single-gene studies remain essential for identifying ISGs and uncovering their molecular mechanisms, additional combinatorial knockout work is needed to pinpoint exactly which set of ISGs target each virus.

Other complex host–pathogen interactions fit this pattern. For example, Legionella pneumophila encodes hundreds of virulence factors but does not deploy them all simultaneously. Instead, it uses a subset tailored to the particular host cell or environmental niche [14]. This parallel underscores a key concept: having a large arsenal does not necessarily mean each component is redundant; rather, it gives the organism—or in the case of interferon, the host immune system—access to distinct sets of effectors suited to different threats. It remains to be determined, however, whether limited sets of ISGs work on most viruses, or whether certain pathogens require more extensive arrays of effectors. For example, DNA viruses with large genomes present a more complicated case, as they encode many immunomodulatory molecules, so interferon-mediated suppression of these viruses may involve a more extensive array of ISGs than that seen with genetically simpler RNA viruses.

The combinatorial knockout experiments described here have revealed a precise, targeted mode of IFN action for an alphavirus and a retrovirus. The next steps will involve broadening these studies to additional viruses and examining how factors like chronic infection or co-evolution shape the selectivity of ISG repertoires. It will also be important to assess this model in vivo, where IFN/ISG responses in complex multi-cellular environments will likely vary considerably when compared to cell culture systems. Additionally, it is tempting to speculate whether targeted approaches that increase the expression of a narrow set of ISGs, via CRISPR activation or gene therapy, for example, could confer antiviral protection without the unwanted side effects of classical interferon-based therapies. Overall, this refined model illustrates a powerful but nuanced antiviral system, one that looks less like a “thousand cuts” and more like a broad array of possibilities, from which only a few antiviral genes are ultimately needed to protect the host.

## Abbreviations:

ISGs: interferon-stimulated genes
VEEV: Venezuelan equine encephalitis virus
